# The effect of vaginal closure technique on early post-operative pain following vaginal prolapse surgery: a feasibility pilot study and qualitative assessment

**DOI:** 10.1186/2193-1801-3-1

**Published:** 2014-01-02

**Authors:** Turlough Maguire, Christopher Mayne, Janet Willars, Douglas Tincello

**Affiliations:** Leicester General Hospital, University Hospitals of Leicester NHS Trust, Gwendolen Road, Leicester, LE5 4PW UK; Department of Health Sciences, University of Leicester, Leicester, UK; Reproductive Sciences Section, Cancer Studies and Molecular Medicine, University of Leicester, Leicester, UK

**Keywords:** Pilot, Surgery, Gynaecology, Prolapse, Sutures, Qualitative

## Abstract

**Background:**

Surgery for pelvic organ prolapse is a common surgical procedure. There is little research studying post-operative pain, contrasting with extensive literature on pain after childbirth trauma which shows that suture method has a significant effect on pain. We designed a protocol for a trial comparing suture techniques and post-operative pain, and conducted a pre-protocol pilot to inform our trial design.

**Methods:**

Routine surgical data, post-operative pain scores (from 10 cm Visual Analogue Scales, VAS) and analgesic requirements were obtained from the notes of a cohort of women undergoing vaginal prolapse surgery. Median VAS scores at 4, 12 and 24 hours were compared by suture material used and method of closure (single continuous suture or interrupted sutures). The women whose data were obtained were invited to attend focus groups of up to six people in the twelve weeks following surgery. A semi structured question guide was used, and interviews were recorded, transcribed anonymously and analysed using the constant comparative method of grounded theory. Ethical approval was not sought because formal pre-protocol work is exempt for UK ethical requirements, but formal written consent on standard forms was obtained for publishing outcomes and anonymised comments from participants.

**Results:**

Complete VAS data and operative details were available from 41 women. Surgery was completed using absorbable polyglactin sutures with continuous suturing (17 women) or interrupted sutures (24 women). Pain scores at 4 and 12 hours were similar; pain at 24 hours was greater in the women with interrupted sutures (median VAS 3 (range 0–8) versus 1.5 (0–8) (p = 0.0513). Analgesic needs were similar.

Two focus groups (nine participants) revealed that women regarded post-operative pain as insignificant and not a topic worthy of formal research. It was apparent that the use, and especially removal of, vaginal packs was a practice associated with pain which women remembered as a significant part of their post-operative experience.

**Conclusions:**

Formal pre-protocol work is informative; we found a moderate difference in our proposed outcomes, suggesting a trial was feasible but women themselves were unconvinced of the need for formal research into pain following vaginal surgery.

## Background

Pelvic organ prolapse is common with up to 20-60% patients attending a menopause clinic having some evidence of uterovaginal or vault prolapse, with 51% anterior wall descent and 27% posterior wall descent (Versi et al. 2001 Handa et al. 2004). The management of prolapse includes conservative measures such as pelvic floor exercises and vaginal pessary supports but surgical treatment remains common and is by no means definitive. There remains a high risk of recurrence up to 29% balanced with a re-operation of 3.7% (Olsen et al. 1997 Maher et al. 2013). Much research has been published examining new techniques of surgery, including mesh, where the primary outcome has been anatomical and symptomatic cure. Post-operative pain after prolapse surgery has been little studied.

In contrast, there are over 1,500 articles studying methods of perineal suturing after childbirth, and the effects upon pain, including two Cochrane systematic reviews. Data from these could be considered analogous and relevant, since they and our research question both address suturing of the vaginal skin and perineum in relation to pain. The first review compared continuous suture techniques versus interrupted sutures for the closure of episiotomies or second degree tears (Kettle et al. 2007). This included seven studies with 3,822 patients and found continuous closure to be preferable with lower pain scores and lesser analgesic requirements. The second follow on review compared different suture material used and included 18 trials with 10,171 patients. Outcomes showed that use of catgut increased short term pain and that rapidly absorbable synthetic sutures (e.g. polyglycolic acid) were preferable to conventional synthetic sutures (Kettle et al. 2010).

We performed a literature review for vaginal surgery and closure techniques performed between 1993–2013 using MEDLINE, EMBASE and CINAHL MeSH heading “sutures” OR “suture” AND “vagina” AND “closure” generating 88 studies none of which looked at techniques for vaginal closure or suture material used. The focus of many of these studies was on closure of the vaginal vault and peritoneum and the effect of these interventions had on the incidence of vault haematoma. Broadening the search to review all studies relating to colporrhaphy with search terms “closure” AND “anterior” OR “posterior colporrhaphy” identified 185 articles over the same time-frame, but of these only one study compared the use of permanent versus absorbable suture in posterior repair and found an increase risk of wound dehiscence and suture erosion with permanent sutures, but no comment on post-operative pain (Luck et al. 2005).

Given the lack of published evidence on the influence of suture material or suture technique on pain after vaginal surgery, we generated the hypothesis that continuous sutures and interrupted sutures cause different amounts of pain, analogous to the data from postnatal perineal repair and we designed a trial protocol to test this hypothesis. As part of this process we conducted a prospective pilot study (on the advice of the East Midlands Research Design service) to measure immediate post-operative pain and to conduct semi-structured interviews with women having surgery to explore their views on the significance and importance of pain in the first few days after surgery.

## Results

43 patients had complete pain VAS data collected over the study period, but two did not have closure technique documented. The remaining 41 women had a mean age of 60 (SD ± 13), median parity of 2 (range 1–5) and BMI of 28.3 (SD ± 4.3). All surgical procedures were carried out using a polyglactin suture material, so the analysis was restricted to type of suture method only. The median pain scores at different time points are shown in Table 1. Pain scores at all times were low (median of 4 or less) and there were no differences between median scores at any time interval between different suture techniques, although the scores at 24 hours (1.5 for continuous sutures vs 3.0 for interrupted sutures) almost reached significance (p = 0.0513). Analgesia use is presented in Table 2 and there were no differences detected in amount or type of analgesia administered.

**Table 1:** **Median visual analogue pain scores; comparing suture methods**

Time	Continuous suture (n = 17)	Interrupted suture (n = 24)	p
4 hours	4 (0–8)	3.5 (0–8)	0.48
12 hours	2 (0–10)	2 (0–6)	0.4331
24 hours	1.5 (0–8)	3 (0–8)	0.0513

Data are median (range).

**Table 2:** **Median analgesia use; a comparison of doses administered**

	Continuous suture	Interrupted suture	p
NSAIDs	2 (0–3)	1(0–3)	0.0762
Paracetamol	4	4 (2–4)	-*
Weak opiate	1(0–4)	2 (0–4)	0.2191
Strong opiate	1(0–4)	2 (0–5)	0.1103

Data are median (range).

NSAID: Ibuprofen/diclofenac/mecoxicam;

Weak opiate – codeine/dihydrocodeine,

Strong opiate – tramadol/morphine sulphate.

* Mann Whitney could not be calculated due to no variation on the data from the continuous suture group.

All 41 women were invited to participate in the focus groups. 12 (29%) agreed to take part, and nine actually attended over two groups. Four themes were identified: pain, the vaginal pack, research and assessment (Table 3). Representative quotations for each theme are presented in the table.

**Table 3:** **Themes identified from focus groups**

Theme	Sample quotes
Pain – the experience	“I think it was more discomfort than real pain”
	“you expect it to be uncomfortable”
	“because I don’t, the word pain to me means something totally different to what I had, and I had discomfort”
	“I think for myself it was more a discomfort than actual severe pain”
	“just a dragging, not really what you’d call pain, just a dragging feeling”
Pain - severity	“Pain means something excruciating, that I can’t stand, that I need something for”
	“well just the degree of discomfort I felt, which I did have, obviously had some discomfort, and I, I only took paracetamol”
Vaginal pack	“I felt a lot better once the pack was out”
	“I can half remember the feeling of it coming out and thinking thank God for that”
	“and when the pack came out obviously it was much more comfortable then, and the, the actual, the sort of the real pain was that I got in, in my back passage”
	“it was more intense and more, like you say, the back passage area, which when I mentioned to the nurse they said oh you will do because of all the stitches and the pack, it’s the pack that’s causing the pressure”
	“a lot better, again once the pack was out and catheter was out, an awful lot better”
	“the most painful thing of all was when they removed the…pack”
Research	“perhaps the pack needs investigating”
	“The thing that seems to cause the most trouble is that pack, that must be essential”
Assessment	“I think, I think it’s difficult to score between one and ten because there’s very little difference say between three and four, so you know, are there too many options”
	“well I think if you said sort of between say one and six, and six was the top, one was the bottom, so you had more idea, are you in the middle or top or bottom”
	“it’s, that’s good, but then if you did, if you had that on one of you leaflets to go home, even if it wasn’t reported back to the hospital, if you could then think to yourself I’ll fill that leaflet in, how am I feeling today, again visually if you can see yourself, how you are”
	“almost need to keep a diary, don’t you?”

The women felt that the sensations experienced after prolapse surgery were not severe enough to warrant use of the word “pain”, preferring to use terms such as “discomfort”, and comparing this favourably to the pain remembered from childbirth, which was regarded as a more “deserving” sensation of the word “pain”. All the women said that pain/discomfort was greatest in the first 24 hours after the surgery often related to the pack (see below) and was well controlled with non-opiate oral analgesia alone.

The vaginal pack appeared to be a strong influence relating to pain in the first 24 hours. All women stated that the pack and its removal was a significant part of their experience and contributed to the discomfort felt in the immediate post-operative period. The significance of this event was clear when women were asked about potential research questions, with the theme relating to vaginal packing re-emerged as a potential area of research, to define whether packing was necessary, since it was the single most important factor related to pain for most of the women.

In contrast, the participants did not feel a research study comparing whether suture methods would have an impact in reducing immediate post-operative pain was an important study. Over half the respondents explicitly stated that they did not think there was a need for research to reduce post-operative pain.

## Discussion

This paper describes a small pilot study to inform the design and feasibility of a planned randomised study comparing suture methods for vaginal prolapse surgery. The findings demonstrate clearly the benefits of pilot work during trial design. The study hypothesis and protocol plan were developed from the limited existing evidence, and from related trial results for suturing after perineal trauma during childbirth. The results clearly showed that while a sound concept, the trial design would not be successful. The outcome data collected suggested a small difference in short term post-operative pain at 24 hours in favour of continuous suturing of the vaginal skin. To confirm a difference of this magnitude would only require 100 women in total, a target which could easily be achieved. However, the women in our focus groups did not consider the sensory experience associated with pelvic organ prolapse surgery was of sufficient intensity to warrant further research looking at pain. Pain is defined as an unpleasant sensory and emotional experience associated with actual or potential tissue damage, or described in terms of such damage (Bonica 1979) and it was notable that most of the respondents chose to use words other than “pain” to describe the sensations experienced.

The median pain scores at 24 hours for continuous technique fell into a low-intensity pain bracket (i.e. <3 on VAS), whereas the interrupted group just fell into a moderate pain intensity group (between 3 and 5 on VAS) (Collins et al. 1997). Given the low rate of analgesia use in either group it is difficult to argue that pursuing a change in technique would have any impact on reducing these analgesic requirements significantly enough to have any health economic benefits.

In contrast to this, the emotional and sensory experience relating to the vaginal pack was an area that women repeatedly raised in our discussions. It was clear that this might represent a topic for further research. Currently there is little research in this area. A Medline search did not identify any publications in relation to vaginal packing for pelvic organ prolapse surgery but we did find three abstracts presented at International Continence Society meetings from 2010–2012. One study randomised 173 women to have a vaginal pack or not, using the McGill pain score as primary outcome (Thiagamoorthy et al. 2010). No difference in day 1 pain score was detected although it was not clear whether this assessment coincided with pack removal, which our data show is the time when pain/discomfort is greatest. The second study randomised 43 women, using complications as primary outcome but found no statistically significant differences in the rate of urinary tract infection or haematoma formation (Baumgarten et al. 2011). The final study allocated 200 women in a non-randomised manner to be packed or not packed, and reported a higher rate of pelvic haematoma formation and vaginal bleeding in the no-pack group with no difference in pain (El Saeed 2012). Thus, there is no convincing evidence for or against routine vaginal packing after prolapse surgery.

Much recent research has examined patient goals after prolapse surgery (Hullfish et al. 2002 Elkadry et al. 2003 Srikrishna et al. 2010 Baskayne et al. 2013) which are dominated by goals concerning relief of symptoms, improved lifestyle, sexual function and emotional health, and concerns that surgery may fail, or new symptoms may arise Concerns about pain have also been raised (Baskayne et al. 2013) but our data suggest that this is a minor issue in the long term, compared to other goals.

We acknowledge that this pilot included a relatively small sample of respondents, although pain outcomes on 41 women were obtained. It is possible the women who attended the focus groups had a different experience of post-operative pain than the whole cohort and we acknowledge this potential for bias. However, despite the relatively small sample size required to confirm the difference in pain score we observed, the lack of enthusiasm for this research question among our respondents was sufficiently large to suggest a trial would struggle to recruit, and would also not provide a clinically meaningful answer, given the low severity of pain scores observed. Thus, the value of patient involvement and mixed methods pilot work for complex interventions (Campbell et al. 2000) is well demonstrated by our data.

## Conclusion

The involvement of patients and public is increasingly seen as an essential part of trial design, and pilot work is invaluable to explore the potential effect size to be expected during a full trial, as well as allowing the exploration of patient views by means of qualitative methods. This small pre-protocol pilot distinctly shows the value of such work within disease areas where quality of life and non-life threatening issues predominate. Our work revealed that a full trial would be unlikely to succeed despite only needing to be of moderate size due to potential participants not identifying the research question as important.

## Methods

We carried out a pilot observational study of all patients undergoing native tissue colporrhaphy for prolapse with or without hysterectomy under the care to two urogynaecologists within our unit over a 9 month period from January to September 2011. Women having mesh-augmented surgery or concomitant incontinence or vault suspension procedures were excluded.

Outcome measures were 10 cm visual analogue scale (Wong-Baker) pain scores recorded routinely and prospectively by the nursing staff caring for each patient at 4, 12 and 24 hours post-operatively. We recorded analgesic requirements in the first 24 hours by reviewing drug charts and prescribed/received medication, and the type of suture and method for closure of the vaginal skin and fascial plication were also recorded. Sutures were classed as non-absorbable (Nylon, Polypropylene) absorbable (polyglactin) and rapidly absorbable (polyglactin-Vicryl Rapide); closure technique was classed as continuous, continuous locking or interrupted. These definitions were made before data collection began, and the analysis was planned accordingly. We did not collect data on operating surgeon, since this was a pilot exercise aimed at obtaining an estimate of pain scores and distribution by the planned primary variable (suture method). There would have been insufficient power to reliably explore surgeon expertise, although this was planned for the main study.

Following the quantitative assessment, all women from the initial study group were sent a postal invitation to participate in focus group sessions. These sessions were aimed at exploring the importance of pain to women undergoing pelvic organ prolapse surgery and to assess feasibility of recruitment into a larger randomised controlled trial looking at vaginal closure techniques. Women were contacted by telephone by one of the researchers and given a verbal explanation of the intentions of the focus group and were invited to attend. On the day those who attended were given time to raise any questions or concerns and then asked to complete a written consent form before the sessions started. Focus groups were assembled to include up to six participants and each session lasted up to two hours. Groups were facilitated by JW and TM using a semi-structured interview approach, with a topic guide which allowed those involved to discuss any areas they felt were relevant. These sessions were audio recorded and transcribed. Analysis was by a grounded theory approach (Patton 1987; Glaser & Strauss , Glaser & Strauss Glaser & Strauss 1967).

The original study protocol for the planned randomised trial was registered on Current Controlled Trials (ISRCTN83130211) in February 2012. Early discussions with the East Midlands NIHR Research Design Service suggested this pilot work to refine the power calculations based on “real world” pain scores, analgesia requirements and some patient perspective on the significance and importance of pain and the research question. This study and analysis was funded by a pre-protocol award from the Research Design service. Pre-protocol work for the purpose of developing and refining a clinical trial study design is exempt from Research Ethics Committee approval (NIHR INVOLVE 2012). However, we provided a written information leaflet in standard REC format and obtained formal written consent from each participant, informing them that we might seek to publish anonymised data and quotations from the focus groups. Collection of routine post-operative pain data, and operation details from the cohort of patients was deemed to be audit by our clinical director so no formal ethics application was made.

## Authors’ information

TM conducted this research while working as subspecialty trainee in Urogynaecology. CJM is a consultant urogynaecologist. JW is a health services researcher with expertise in qualitative interviews. DGT is professor of Urogynaecology and a consultant urogynaecologist. He has experience in the design and conduct of clinical trials in Urogynaecology.

## Abbreviations

VAS: Visual analogue scale.
